# A novel genetic locus linked to pro-inflammatory cytokines after virulent H5N1 virus infection in mice

**DOI:** 10.1186/1471-2164-15-1017

**Published:** 2014-11-24

**Authors:** Adrianus CM Boon, Robert W Williams, David S Sinasac, Richard J Webby

**Affiliations:** Departments of Internal Medicine, Division of Infectious Diseases, Molecular Microbiology and Pathology and Immunology, Washington University School of Medicine, St Louis, MO 63110 USA; Department of Infectious Diseases, St. Jude Children’s Research Hospital, Memphis, TN 38105 USA; Department of Anatomy and Neurobiology, University of Tennessee Health Science Center, Memphis, TN USA; Department of Medical Genetics, University of Calgary, Calgary, Alberta Canada

**Keywords:** H5N1 influenza virus, BXD, Quantitative trait locus analysis

## Abstract

**Background:**

Genetic variation in the human population is a key determinant of influenza disease severity. A single nucleotide polymorphism in the antiviral gene *IFITM3* was linked to outcomes during the 2009 H1N1 pandemic. To identify variant host genes associated with increased virus replication and severe disease, we performed a quantitative trait locus analysis on pro-inflammatory cytokine production 48 hours after intranasal infection with highly pathogenic H5N1 influenza virus.

**Results:**

Pro-inflammatory cytokines CCL2, TNFα and IFN-α, were measured by ELISA in lung homogenates of DBA/2J (D2), C57BL/6J (B6) and 44 different BXD recombinant inbred mouse strains. Virus titer was also assessed in a subset of these animals. CCL2 (8-fold), TNFα (24-fold) and IFN-α (8-fold) concentrations varied significantly among the different BXD RI strains. Importantly, cytokine concentration correlated very well (r =0.86-0.96, P <0.0001) with virus titer suggesting that early cytokine production is due to increased virus infection and replication. Linkage analysis of cytokine concentration revealed a significant locus on chromosome 6 associated with differences in TNFα, IFN-α and CCL2 cytokine concentration (LRS =26). This locus accounted for nearly 20% of the observed phenotypic variation in the BXD population studied. Sequence and RNA expression analysis identified several candidate host genes containing missense mutations or deletions; *Samd9l*, *Ica1*, and *Slc25a13*. To study the role of *Slc25a13*, we obtained *Slc25a13* knockout line, but upon challenge with H5N1 influenza virus observed no effect on CCL2 production, or morbidity and mortality.

**Conclusion:**

A novel genetic locus on chromosome 6 modulates early pro-inflammatory cytokine production and virus replication after highly pathogenic influenza virus infection. Candidate genes, *Samd9l* and *Ica1*, may be important for the control of influenza virus infection and pathogenesis.

**Electronic supplementary material:**

The online version of this article (doi:10.1186/1471-2164-15-1017) contains supplementary material, which is available to authorized users.

## Background

Severe respiratory and systemic disease caused by human infection with avian H5N1 influenza virus is characterized by high viral load and increased production of proinflammatory cytokines, which if left untreated, often results in death [1, 2]. Epidemiological studies of outbreaks of the highly pathogenic H5N1 virus in Asia suggest that human genetic polymorphisms influence disease severity [3, 4]. More recently a single nucleotide difference in the antiviral *IFITM3* gene was associated with hospitalization and more severe disease after the pandemic H1N1 influenza infection [5].

Pathogenesis after influenza virus infection is the result of a complex interaction between the virus, immunity, and environment [6–8]. The early events after infection are critically important for the progression and outcome of the disease [9, 10]. Lack of virus neutralizing antibodies or genetic variation in the virus [10] or the host [11] can increase early virus replication and over-stimulate the innate immune response creating a pathogenic host response. The inflammatory environment associated with a pathogenic response can blunt the adaptive immune response [10] and promote virus replication through recruitment of susceptible cells [12]. Identifying the viral and host genetic determinants that contribute to alterations in early replication and ensuing host response is important for the control of pathogenesis and the identification of targets for the design of novel therapeutics. Forward genetic studies in mice have identified several genetic factors that contribute to survival after infection [13–16], but few have studied the very early host response after infection [15].

Infections with H5N1 and H7N9 are highly pathogenic and cause severe morbidity and mortality. To identify host genes and gene polymorphisms critical for the differences during the early stages of H5N1 disease we have performed a QTL analysis on genetically diverse strains of mice looking at the production of pro-inflammatory cytokines CCL2, TNF-α, and IFN-α, 48 hours post infection. We identified a single genetic locus on chromosome 6 containing several polymorphisms associated with an acute increase in the production of pro-inflammatory cytokines. The identification of this locus and several intriguing candidate genes suggests that the host cytokines response early after infection is due to a limited number of genetic polymorphism.

## Results

### Differences in pro-inflammatory cytokine production among mouse strains

**Figure 1:**
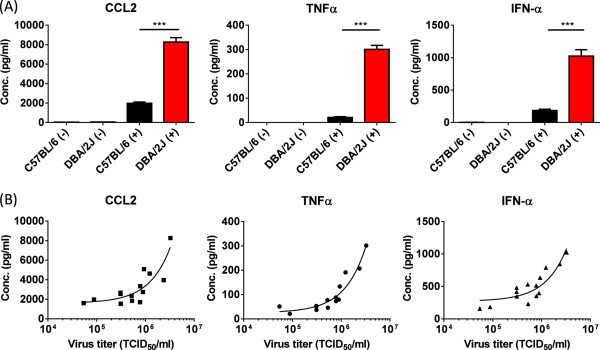
**Increased concentration of CCL2, TNFα, and IFN-α in lung homogenates of A/Hong Kong/213/03 H5N1 virus infected DBA/2J mice. (A)** DBA/2J (D2) and C57BL/6J (B6) were inoculated with 10^4^ EID_50_ of HK213 virus (+) or mock-inoculated (-) in 30 μl PBS. Forty-eight hours post inoculation the lungs of the inoculated animals were collected, homogenized in sterile PBS, and stored at -80°C. The concentration of CCL2, TNFα, and IFN-α in these homogenates was quantified by ELISA. The average cytokine concentration (pg/ml ± standard error of the mean) of CCL2, TNFα and IFN-α is shown for mock-infected (n =4, single experiment), C57BL/6 (n =13 from 4 different experiments), and DBA/2J (n =15 from four different experiments). *** is P <0.0001. **(B)** Significant association between CCL2, TNF-α, and IFN-α concentration and lung virus titer 48 hours post inoculation with 10^4^ EID_50_ of HK213 virus in B6, D2 and BXD recombinant inbred mouse strains (P < 0.0001 for all three cytokines).

Genetically diverse mice vary greatly in response to influenza virus infection and we think this is the result of higher virus replication in the first 24–72 h after inoculation. The increase in viral titers in the lungs of susceptible strains, such as D2, leads to the increased production of pro-inflammatory cytokines compared to resistant strains, such as B6 [11, 13]. To understand the genetic and molecular basis for this difference in host response and early viral load, we quantified inflammatory host response 48 hours after inoculation with 10^4^ EID_50_ of an H5N1 virus (HK213). This time point coincided with peak viral replication [13] and the earliest point at which we can reliably measure the production of IFN-α (Additional file 1: Figure S1). To quantify the inflammatory response after HK213 infection we measured titers of CCL2, TNFα and IFN-α in whole lung homogenates. Compared to mock-infected animals the concentration of CCL2, TNFα, and IFN-α increased significantly (P <0.0001, Figure 1) in both parental strains after inoculation. More importantly, levels of CCL2, TNFα, and IFN-α were significantly higher in D2 compared to B6 (P <0.001 for all three cytokines), confirming our previous studies suggesting that early cytokine levels correlate with virus titer.To evaluate this further we quantified the lung virus titer as well as the CCL2, TNFα, and IFN-α concentration in 12 different BXD RI mouse strains (Figure 1B) 48 hours post inoculation. Correlation analysis revealed a highly significant association between lung virus titer and TNF-α (r =0.96, P < 0.0001), CCL2 (r =0.86, P < 0.0001), and IFN-α concentration (r =0.88, P <0.0001) at 48 hours post inoculation.

### Identification of candidate genomic loci associated with increased levels of pro-inflammatory cytokines

**Figure 2:**
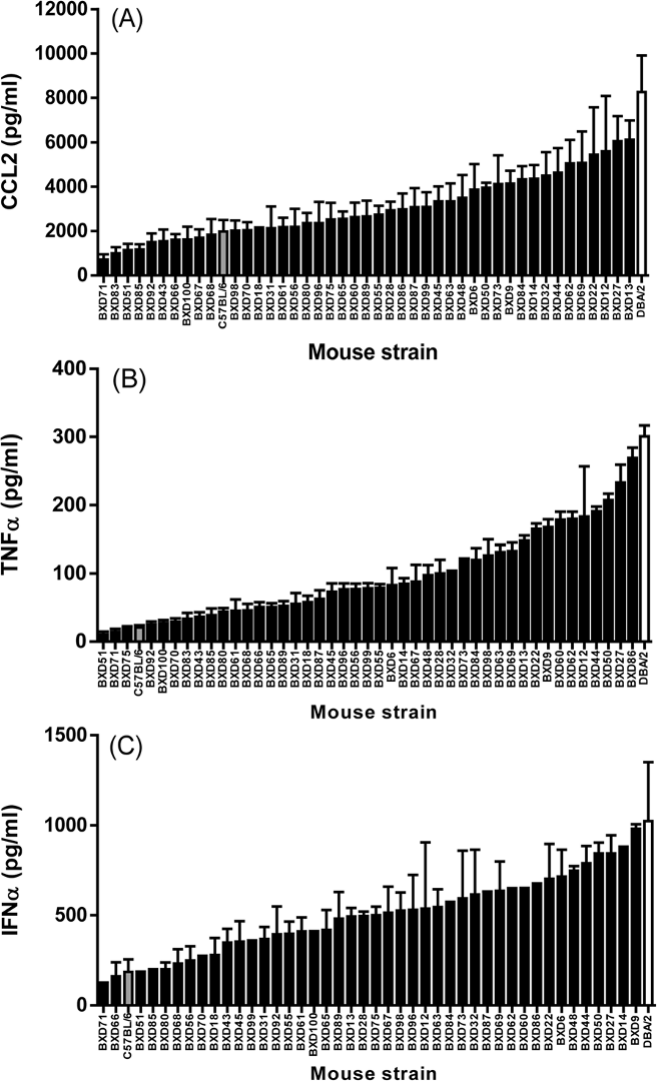
**CCL2, TNFα, and IFN-α concentration in lung homogenates of BXD recombinant inbred mouse strains.** Forty-four different BXD RI mouse strains (derived from DBA/2J and C57BL/6) were infected with 10^4^ EID_50_ of HK213 influenza virus in 30 μl PBS. Forty-eight hours post inoculation the lungs of the inoculated animals were collected, homogenized in sterile PBS, and stored at -80°C. The concentration of CCL2, TNFα, and IFN-α in these homogenates was quantified by ELISA. The average cytokine concentration (pg/ml ± standard error of the mean) of CCL2 **(A)**, TNFα **(B)** and IFN-α **(C)** is shown for each BXD RI strain (black bars) and the parent strains (DBA/2J (white bar) and C57BL/6 (grey bar)). The average cytokine concentration for each BXD strain was calculated from 2-10 animals (average =5.2) and 36% of the BXD strains have been repeated independently.

The concentrations of TNFα, CCL2 and IFN-α in the lungs of HK213 infected BXD RI mice ranged from 11-269 pg/ml, 728-6128 pg/ml, and 127-982 pg/ml respectively (Figure 2 and Additional file 2: Table S1). Interestingly, several BXD strains produced equally low amounts of CCL2, TNFα, and IFN-α as the parent B6. In contrast, none of the BXD strains produced similarly high levels of cytokines as the D2 parent, suggesting that the high cytokine-producing phenotype of D2 had reached a maximum asymptote dependent on the cumulative effects of several sequence variants. Regression analysis identified a highly significant correlation between CCL2 and TNFα (*r =*0.82, P <0.0001), CCL2 and IFN-α (*r =*0.78, P <0.0001), and TNFα and IFN-α (*r =*0.81, P <0.0001) concentrations in lung homogenates, suggesting a common mechanisms modulating levels of cytokine production among BXD strains.

**Figure 3:**
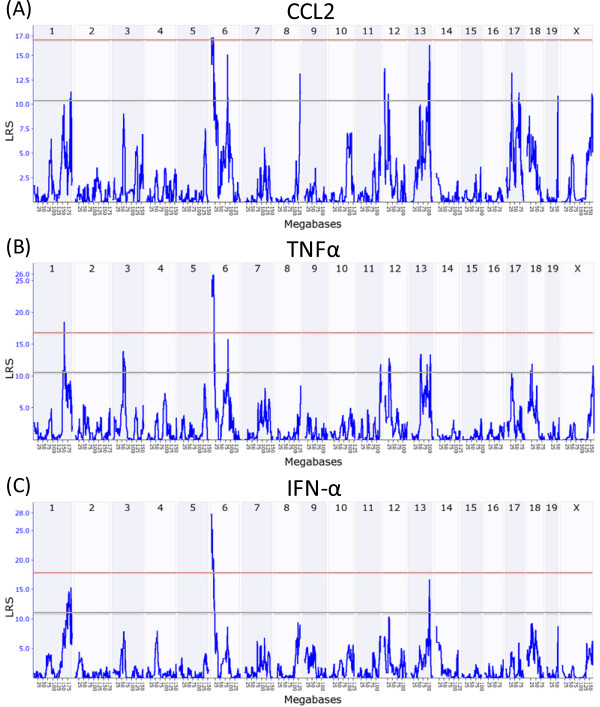
**Genome-wide linkage analysis CCL2, TNFα, and IFN-α production after H5N1 virus infection.** QTL analysis (www.genenetwork.org) was performed on the CCL2 **(A)**, TNFα **(B)**, and IFN-α **(C)** cytokine concentrations in homogenates of lungs 48 hours after infection with HK213 H5N1 influenza virus. QTL associated with increased CCL2, TNFα, and IFN-α concentration after infection with HK213 virus (*Qivr6.1*) was identified on chromosome 6 (3.4 - 14.5 Mb) as indicated by an LRS score of more than 17.7 (pink horizontal line in QTL plot). A second QTL associated with increased TNFα concentrations was located on chromosome 1 (*Qivr1*, 154.5 - 160.6 Mb). This same locus also reached suggestive P-values for IFN-α. Suggestive loci were also found for CCL2 and TNFα on chromosome 6 (*Qivr6.2*, 81 - 89 Mb) and for IFN-α and CCL2 on chromosome 13 (*Qivr13*, 103.2 - 107.2 Mb. Pink line denotes significant linkage (genome-wide P <0.05, LRS >17.7) and grey line indicates suggestive linkage (LRS >12). For this study we focused on suggestive loci that were nearly significant and identified in two or more pro-inflammatory cytokine QTL analysis.

QTL analysis on cytokine profiles identified a highly significant locus located on chromosome 6 between 3.4 Mb and 14.5 Mb (Figure 3). This locus, *Qivr6.1*, is associated significantly (P-genome wide <0.05) with the amount of TNFα (peak LRS =25) and IFN-α (peak LRS =27) produced 48 hours after intranasal infection with HK213. *Qivr6.1* also accounts for variation in the concentration of CCL2 in lung homogenates, albeit at a lower level of significance (peak LRS =17). In general, *Qivr6.1* accounted for ~18% of the phenotypic difference in cytokine levels among BXD strains and the D2 allele increased the trait value. Winsorization of the data confirmed the genetic association for *Qivr6.1*. In addition to the highly significant locus on the proximal part of chromosome 6, we also identified a suggestive locus (*Qivr6.2*) for TNFα and CCL2 on the distal portion of chromosome 6 between 81 and 89 Mb (peak LRS =15). Another suggestive locus was identified for IFN-α and CCL2 on chromosome 13 (*Qivr13*) between 103.2 and 107.2 Mb (peak LRS =16). Finally, TNFα concentrations associated significantly with a locus on chromosome 1 (*Qivr1*) between 154.5 and 160.6 Mb (peak LRS =17).

### Identification of candidate genes in Qivr6.1

To identify the genetic polymorphisms and host genes that are responsible for the difference in production of pro-inflammatory cytokines after HK213 virus infection, we queried available sequence information for genetic polymorphisms between 3–16 Mb on chromosome 6. Three genes—*Samd9l*, *Slc25a13*, and *Ica1*—contain non-synonymous SNPs, and one gene—*Col28a1*—has a large in-frame deletion spanning three coding exons (exon 16 through 18 in NM_001037865.1) the D2 genotype (Table 1). All four genes are expressed in HK213-infected lung tissue of D2 and B6 strains and we confirmed these mutations by sequence analysis of PCR products using gene-specific primers and RNA extracted from HK213-infected lung tissue from D2 and B6 (see Additional file 3: Table S2 for primer sequences). In addition to these candidate genes, we also performed Sanger sequencing on *Pon1*, *Pon2*, *Pon3*, *Hepacam2*, *Cdcc32*, *Asb4*, and *C1galt1* because of their potential to affect cytokine production or alter influenza virus infection. The reported single nucleotide polymorphisms in *Samd9l* (rs30759164 (V_169_I) and rs30516773 (I_459_V)), *Slc25a13* (rs32512230 (V_583_A) and rs30695207 (A_648_T)), and *Ica1* (rs51311141 (A_312_T) and rs6218746 (Q_397_R)) were confirmed by sequencing. Similarly, we also confirmed the in-frame 69 amino-acid deletion in *Col28a1* in the D2 genotype. No additional missense or nonsense polymorphisms or deletions and insertions were identified between D2 and B6 in the coding region of any of the other seven host genes that were targeted for sequencing.

**Table 1:** **Missense mutation analysis across multiple inbred mouse strains**

Gene	SNP	DBA/2J	129/SvImJ	A/J	SM/J	C57BL/6J	Balb/cJ
***Samd9l***	V_169_I	I	I	I	I	V	I
	I_459_V	V	V	V	V	I	V
***Slc25a13***	V_583_A	A	V	V	V	V	V
	A_648_T	T	A	A	nd^1^	A	A
***Ica1***	A_312_T	T	T	T	T	A	A
	Q_397_R	R	R	R	R	Q	Q
***Col28a1***	Deletion^*^	Del	-^2^	Del	-	-	-

*Based on two flanking non-synonymous SNP (rs30133088 at position 312 and rs30660613 at position 602). ^1^ nd = no data available. ^2^ = similar to wild type C57BL/6.

Next, we evaluated the missense polymorphisms across several other inbred strains of mice that were previously characterized for their response to influenza virus (Table 1) [5]. The missense mutations in *Samd9l* and *Slc25a13* were unique to B6 and D2 strains respectively. In contrast the genetic differences in *Ica1* segregated well between resistant and susceptible mouse strains. The exception was SM/J whose genome harbored the susceptible missense mutations in *Ica1*. The presence of the 69 amino-acid deletion in *Col28a1* was also present in the A/J strain of mice, but not in the susceptible 129/SvImJ or the resistant mouse strains.

### RNA expression analysis of candidate genes in Qivr6.1

Using previously published RNA expression data [13] from lungs obtained from D2 and B6 mice infected with H5N1 influenza virus, we identified 41 host genes within *Qivr6.1* that were detected by microarray in the context of uninfected or H5N1 IAV infected lung tissue. These included the three polymorphic host genes *Samd9l*, *Ica1* and *Slc25a13*, but not *Col28a1*. A comparison of baseline RNA expression identified 5 host genes whose expression levels were significantly different (P <0.01), albeit the difference was less than 2-fold. These included *Ica1*, *Col1a2*, *Calcr*, *Tfpi2* and *Bet1*. One probe (1431380_at), detecting a transcript located within an intron of *Ica1*, was detected only in B6 animals. Within the locus we also identified host genes whose expression increased (*Samd9l*, *Asns*, *Gpr85*, and *Tfpi2*) or decreased (*Pon1*, *Hepacam2*, *Tmem106b*, and *Pppr9a1*) 2-fold (P < 0.05) 72 hours after infection with H5N1 IAV in D2, B6 or both mouse strains. Importantly we did not identify a transcript that was differentially expressed only in D2 or B6 mice suggesting that there are no sequence polymorphisms in important transcription factor binding sites.

Finally, we evaluated available lung micro array data from the QTL gene-network website, correlating TNF-α cytokine levels 48 hours after H5N1 IAV infection with basal gene expression in 37 BXD mouse strains [17]. Interestingly, *Ica1*, *Samd9l*, *Tfpi2* and *Calcr* correlated well (Spearman Rank order correlation >0.4 or < -0.4, P < 0.01) with TNF-α cytokine concentration in these mice.

### Role of Slc25a13 on cytokine production and pathogenesis after H5N1 infection

**Figure 4:**
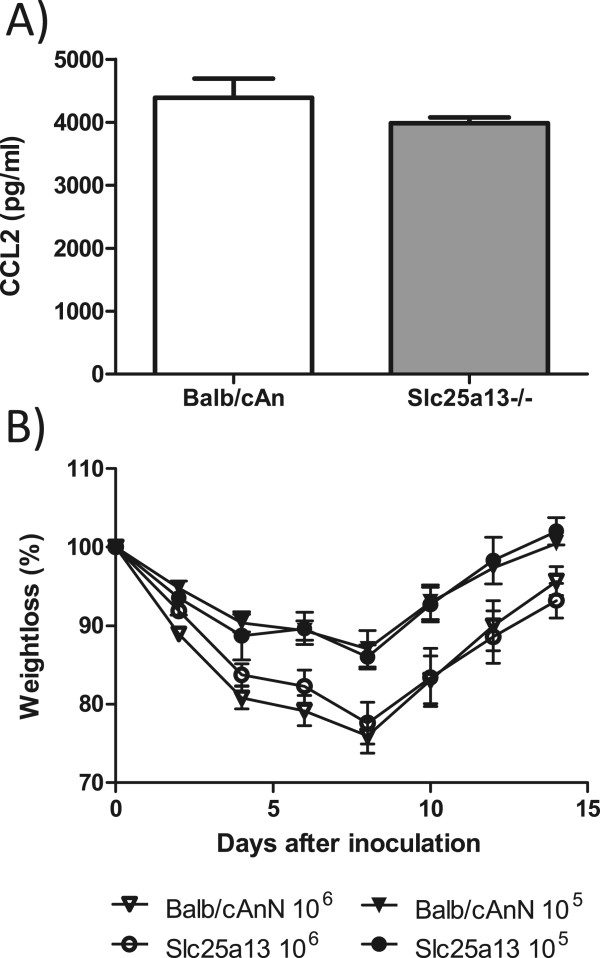
**Effect of** ***Slc25a13*** **gene expression on H5N1 influenza virus pathogenesis. (A)** Slc25a13-deficient mice (*Slc25a13* ^-/-^) and control animals were inoculated with 10^5^ HK213 virus for 48 hours and the amount of CCL2 produced in the lungs was measured by ELISA. The results are from 5 mice per group and are representative of two experiments. **(B)** *Slc25a13* ^-/-^ mice (circles, n =7) and control animals (triangles, n =9) were infected with 10^5^ (filled symbols) or 10^6^ (open symbols) EID_50_ of a highly pathogenic H5N1 influenza A virus (A/Hong Kong/213/03 (HK213)) and weight-loss was monitored for 15 days.

Citrin (the protein product of *Slc25a13*) is a member of the mitochondrial carrier family of proteins and catalyzes the calcium-dependent exchange of cytoplasmic glutamate with mitochondrial aspartate across the mitochondrial inner membrane [18–20]. Mutations in this gene result in adult-onset type II citrullinemia (OMIM 605814) and neonatal intrahepatic cholestasis caused by citrin deficiency (OMIM 603471) that lead to distinct and overlapping effects on hepatic function through its role in urea cycle function [21]. Because citrin was previously reported to interact with the PA polymerase protein of influenza A virus [22] and a gene-knockout mouse was available we decided to test the hypothesis that *Slc25a13* controls the level of cytokine production. Therefore we predicted that the absence of this gene would increase cytokine production upon HK213 infection. To control for the appropriate genetic background we used BALB/cAnCrl as controls for the *Slc25a13*^-/-^ knockout mice congenic on the BALB/cAnN background. Compared to HK213 infected control animals, the *Slc25a13*^-/-^ animals produced equal amounts of CCL2, suggesting that the lack of *Slc25a13* expression did not affect H5N1 induced inflammation (Figure 4A). With respect to disease severity, we monitored weight loss and mortality over a 15 day period after inoculation with 10^5^ or 10^6^ EID_50_ of HK213 virus. No effect of *Slc25a13* gene expression on morbidity (Figure 4B) or mortality (data not shown) was observed.

### Potential role for Samd9l, Ica1 and Col28a1 in influenza virus infection and pathogenesis

Here we have identified a highly significant and novel locus on the extreme proximal portion of chromosome 6 (*Qivr6.1*) that harbors three candidate genes associated with increased inflammation early after H5N1 IAV infection. *Samd9l* is a Sam domain containing protein with similarities to *SAMD9* which has been shown to be important during virus infection and innate immunity [23]. *Samd9l* is expressed in many different tissues including lung [24] and is upregulated by type I interferon. Knockdown of *Samd9l* expression increases West-Nile virus replication [25], although the mechanism is unknown. *Samd9l* knockout mice are characterized by a delay in homotypic endosome fusion resulting in persistence of ligand-bound cytokine receptors [26]. IAV enters cells via the endosomes requiring low pH to trigger membrane fusion and deposition of its genomic material into the cytoplasm. If *Samd9l* is important for the trafficking, maturation and acidification of the endosome, it may act as an antiviral host gene against IAV infection. Interferon induced transmembrane protein 3 (*IFITM3*) is a host protein with potent antiviral activity. *IFITM3* blocks entry of many difference viruses, including influenza, at the stage of membrane fusion in the endosome. Deletion of *Ifitm3* in C57BL/6 mice resulted in increased virus load in the lungs and increased morbidity and mortality after influenza virus infection [27]. While there are no non-synonymous polymorphisms in the *Ifitm3* protein between the D2 and B6 mice, the increase in viral load and associated disease in *Ifitm3*^-/-^ mice is similar to the phenotype observed in D2 mice. An alternative hypothesis is that *Samd9l* affects the persistence of ligand-bound cytokine receptors which may increase cytokine signaling and inflammation which in turn results in more recruitment of inflammatory cells and virus infection [12]. Although two non-synonymous amino-acid changes at position 169 and 459 in the BXD family are predicted to have minimal effect on protein activity (SIFT prediction program), the reported antiviral activity of *Samd9l* against another RNA virus [25] and its effect on endosome function make *Samd9l* our priority candidate gene.

*Ica1* is another candidate gene. This islet cell auto antigen that is the target of auto antibodies in type I diabetes and Sjögren’s syndrome [28]. *ICA1* was also identified in a GWAS looking at genetic modifiers of glaucoma [29]. *Ica1* together with *Pick1* localizes to the trans-Golgi network (TGN) and is involved in the regulation of secretory vesicle trafficking [30, 31]. *Ica1* is highly expressed in nervous, glandular and muscle tissue and to a lesser extend in other tissues such as the respiratory tract. Differences in *Ica1* protein structure or expression can affect the sympathetic nervous system activity and neuropeptide secretion altering the host response to infection [32–34]. Changes in vesicle trafficking may also alter mucus or antibody production reducing innate immune defenses to the virus and allowing for enhanced replication and increased cytokine production. *Ica1* could potentially affect the virus-encoded matrix 2 (M2) ion channel activity in the TGN to neutralize the acidic pH of the TGN in order to prevent activation of HA to its fusogenic form. This activity activates NLRP3 resulting in the formation of the inflammasome, cleavage of pro-IL-1β [35, 36] and increased inflammation. Finally, *Ica1* may alter the replication kinetics of IAV by changing intra-cellular trafficking of TGN-associated vesicles required for trafficking of the viral glycoproteins HA and NA to the surface of the cells. Interestingly the amino acid residue at position 312 in the C57BL/6J strain was predicted to be damaging (SIFT functional polymorphism prediction program), suggesting that the *Ica1* allele in DBA/2J mice may enhance virus replication.

Finally, Col28a1 is a von Willebrand factor A domain-containing protein with many imperfections in the collagenous domain [37]. The 69 amino acid deletion spans one of the many collagen triple helix repeat domains of the protein and it is not clear how this affects protein activity. Interestingly, Col28a1 contains a serine protease inhibitor domain, which could be important in reducing inflammation or virus infection [38]. A polymorphism in COL28A1 was associated with the cytokine response to smallpox vaccination [39].

## Discussion

Morbidity and mortality after influenza virus infection is affected by genetic variation of important host genes. Identification of the polymorphism and affected host genes will provide significant insight into the pathogenesis after influenza virus infection. In order to identify host genes with important effects on influenza pathogenesis, we performed a quantitative trait locus analysis on genetically diverse mouse strains comparing the early inflammatory response after H5N1 virus infection. This analysis identified a highly significant H5N1 control locus (LRS 26) on the extreme proximal part of mouse Chr. 6 that is homologous to a 5 megabase interval on human Chr. 7p22 and 7q21 respectively.

Severe disease after influenza virus infection is mediated by both host and viral factors and many studies suggest that viral load and rapid replication are the determining factor for the outcome of the disease. These findings are supported by potent effects of neutralizing antibodies at early time points and the relatively short period of time (48 hours) in which treatment with antiviral therapeutics is considered most effective. In contrast to these studies, several others suggest that inflammation is independent of viral load and caused by underlying illnesses, or differences in viral protein function, essentially changing the host response to infection from protective (disease prevention) to pathogenic (disease promoting). Interferon antagonist functions of NS1 or the endonuclease function of PA-X are important examples of this process [40]. This view is supported by studies using immune-modulatory agents, like peroxisome proliferator-activated receptor-gamma agonist, lipid mediators or sphingosine-1-phosphate receptor agonists that reduce inflammation without changing the viral load [41–43]. More recently it was reported that inflammation itself is promoting virus replication by recruiting cells to the site of infection allowing the virus to grow to higher titers [12]. While many of these findings are not mutually exclusive, our model using different inbred mouse strains favor the explanation that elevated virus replication and infection are inducing the increase in inflammatory response and subsequent excessive mortality in the susceptible mouse strains. We now provide additional evidence that inflammation and virus titers correlate significantly (P < 0.0001) across a subset of BXD RI lines with correlation coefficients between 0.86 and 0.96.

A major advantage of using inflammatory cytokines as a biomarker for viral load is availability of commercially available and validated assays and the increased dynamic range between the different mouse strains. Although we observed a 50-fold difference in viral titers between D2 and B6 parental strains, on a logarithmic scale this difference is much smaller (1.4-fold). In contrast, the cytokine data is presented on a linear scale greatly increasing our dynamic range between the B6 and D2 animals while maintaining large fold differences in cytokine concentrations between D2 and B6. Thus a QTL analysis with cytokine concentration as the phenotype is more sensitive and likely to yield loci containing genetic polymorphisms involved in virus replication or antiviral activity. Although we provide evidence that inflammatory cytokine concentration in lung homogenates can be used as a surrogate biomarker for viral load between different mouse strains, extensive validation in nasal washes or serum from human patients infected naturally or experimentally with influenza virus needs to be done before this can be used in clinical specimens.

Several loci and polymorphisms have been associated with resistance to influenza virus disease using a variety of different genetic mouse models. BXD RI strains were used to identify genetic loci (*Qivr* or quantitative trait for influenza virus resistance) on chromosomes 2, 5, 7, 16, 17 and 19 [13, 15]. The collaborative cross, which includes B6 but not the D2 mouse strain, was used to identify four different “host response to influenza” virus loci (*HrI*) located on chromosomes 1, 7, 15, and 16 [16]. Finally, BXA/AXB strains were used to map loci on chromosome 2, 4, 6, and 17 associated with more severe influenza virus infection [14]. The loci reported in this study have not been identified previously despite the repeated use of BXD strains. Differences in phenotype, from very early (inflammation 48 hours after infection) to late (survival), and choice of mouse and virus strains will identify different genetic polymorphisms. The current study identified several other loci associated with increased production of pro-inflammatory cytokines after H5N1 influenza virus infection. The locus on chromosome 1 (*Qivr1*), associated with increased levels of TNFα, contains a well-known host antiviral gene ribonuclease L (*Rnasel)*. The presence of at least sixteen different missense mutations between B6 and D2 (including three that are predicted damaging) make this gene a likely candidate. *Qivr13* contains four host genes harboring non-synonymous polymorphisms including *Erbb2ip,* a regulator of Nod2-dependent NF-kappaB signaling, and a member of the nuclear importin family (*Ipo11)*. Finally *Qivr6.2* contains several polymorphic host genes including *Mxd1* and *Anxa4*.

To identify candidate genes we focused primarily on sequence variation that affects the protein (non-synonymous polymorphisms). However sequence variation in regulatory regions of the gene, such as transcription factor binding sites or micro-RNA target sites, can change the level of protein expression and as such impact virus infection and inflammation. At least for *Qivr6.1*, we did not observe large differences (>2-fold) in baseline expression of host genes nor did we identify lack of induction of host genes in the susceptible D2 strain. Therefore we consider it unlikely that sequence variation in regulatory or non-coding regions of the genes present in this locus is causing the difference in phenotype.

*Slc25a13* was one of four candidate genes within the *Qivr6.1* locus associated with increased cytokine production following H5N1 IAV infection. However, mice lacking this gene did not demonstrate a change in cytokine production, suggesting that *Slc25a13* is not the host gene whose polymorphism affected the early innate immune response. Previously, *Slc25a13* was found to associate with the PA gene of a related highly pathogenic H5N1 influenza virus (A/Vietnam/1203/04) [22]. The absence of this interaction in the *Slc25a13*^-/-^ had no discernable effect on the host response or pathogenesis *in vivo*. Alternatively the effects of *Slc25a13* gene deletion vary between mouse strains. We tested the effect of *Slc25a13* on a Balb/cAn genetic background that is different from both parental strains used to generate the BXD family. It is feasible that the deletion of *Slc25a13* in C57BL/6J or DBA/2J mice would change the cytokine profile after H5N1 virus infection. Deletion of the *Slc25a13* gene was previously reported to have variable effects on metabolism dependent on the genetic background of the mouse strains [44]. Alternatively, the deletion of *Slc25a13* may not mimic the effects of the genetic changes found in the D2 strain. The amino-acid changes may have altered gene function that is not captured in the *Slc25a13* knockout mouse strain.

In conclusion, gene polymorphisms in the genome of the infected host can have a profound impact on the course of an H5N1 influenza virus infection. Although severe disease outcomes are often attributed to inflammation with excessive production of proinflammatory cytokines, the pathogenicity of influenza infection is rooted in increased virus titers in the lungs of the susceptible hosts.

## Conclusions

Severe disease after influenza virus infection is associated with excessive production of inflammatory cytokines and recruitment of innate immune cells into the infected tissue. An increase in dose or enhanced virus replication contributes to the excessive production of inflammatory cytokines. Identification of host-factors that facilitate virus replication and pathogenesis is important for the discovery of host targets for the development of novel antiviral therapies.

In this report we provide further evidence that excessive production of inflammatory cytokines is due to higher virus loads. We also show that pro-inflammatory cytokine production following infection with highly pathogenic H5N1 influenza virus is dependent on the genetic background of the mouse strain. Quantitative trait locus analysis identified a locus on chromosome 6 that is associated with early production of pro-inflammatory cytokines and increased virus replication in BXD recombinant inbred mouse strains. Expression and coding sequence analysis identified *Samd9l* and *Ica1* as strong candidate genes in this locus.

## Methods

### Mouse strains and influenza virus

Female DBA/2J (D2, stock no. 000671) and C57BL/6J (B6, no. 000664) mice were purchased from the Jackson Laboratory (Bar Harbor, ME) and housed at St Jude Children’s Research Hospital, Memphis, TN, USA. Female BXD recombinant inbred (RI) mouse lines derived from crosses between B6 and D2 stock were acquired from Oak Ridge National Laboratory (ORNL, Oak Ridge, TN). Mice deficient in citrin (*Slc25a13*^tm1Lct^), congenic on a BALB/cAnN genetic background, were obtained from the Jackson Laboratory [45]. These mice were originally described by Dr. David Sinasac as an animal model for type II citrullinemia. Suitable control animals, BALB/cAnNCrl (no. 028), were obtained from Charles Rivers Laboratories. All mice were between 6 and 10 weeks of age. Infection studies in mice were conducted under the approval of the SJRCH Institutional Animal Care and Use Committee.

A highly pathogenic H5N1 influenza A virus was created by reverse genetics as described previously [46, 47]. The virus contains seven gene segments of A/Hong Kong/213/2003 H5N1 virus and the *PB1* gene segment from A/Chicken/Hong Kong/Y0562/2002 H5N1 virus. This highly pathogenic H5N1 virus, referred to as HK213, was also used in a previously study of pathogenesis in BXD strains [13].

Mice were inoculated with IAV intranasally in 30 μl of sterile PBS after sedation with Avertin (2,2,2-tribromoethanol, Sigma-Aldrich, MO, USA). To assess morbidity and mortality after HK213 virus infection, animals were weighed every second day until 21 days post-infection. Animals that lost more than 30% of their initial body weight were sacrificed per approved animal protocol.

### Lung virus titer and cytokine analysis

Lungs from B6, D2, and BXD strains infected with 10^4^ EID_50_ were obtained 2 days after infection and immediately homogenized in 2.0 ml tubes containing 1.0 ml PBS and a stainless steel ball for two 30-sec periods at 30 Hz (TissueLyzer, Qiagen). After a 30-sec spin at 16,000 × *g*, the supernatant was collected, aliquoted, and stored at –80°C. Virus titers were determined on MDCK as described previously [13]. The concentrations of chemokine (C-C motif) ligand 2 (CCL2) and tumor necrosis factor-alpha (TNFα) in the lung homogenates of infected animals were determined using Quantikine kits from R&D Systems (Minneapolis, MN). The concentration of interferon (IFN)-α was determined with the IFN-α ELISA kit from PBL laboratories (Piscataway, NJ). The average values for each BXD strain represent data from 2 to 10 cases per strain (average =5.2) with 16 of the 44 BXD strains repeated independently (Additional file 2: Table S1). The data for D2 and B6 are the average of 13 and 15 cases, respectively, obtained from four independently repeated experiments. To control for inter-ELISA assay variation, a fresh aliquot of pooled lung homogenates of HK213 virus infected D2 and B6 mice was analyzed in each cytokine ELISA. The values for CCL2, TNFα, and IFN-α in the pooled sample varied no more than 15% from the average of all performed assays.

### QTL mapping

Quantitative trait loci (QTL) mapping was performed using the WebQTL module of GeneNetwork (http://www.genenetwork.org). Interval mapping evaluates a potential QTL at regular intervals and estimates the significance at each location by using 2000 or more permutation tests [48, 49]. Mapping was done using data from 44 BXD RI strains for CCL2 and TNF-α and 43 strains for IFN-α concentration in whole lung homogenates after infection with H5N1. For the original data set, see http://www.genenetwork.org (ID numbers 12971–12973). Loci associated with increased production of pro-inflammatory cytokines were identified and are referred to here as *Qivr* (QTL for influenza virus resistance). The QTL boundaries were defined as a drop in peak LRS of 7 as this is considered the best approximation of a 95% confidence interval in QTL mapping [50]. To reduce the potential effects of outliers on the mapping outcome we also performed the QTL analysis after winsorization; a technique to reduce the effects of outliers on statistical analysis [51]. Winsorization did not affect the outcome of the analysis and the same significant and suggestive loci were identified. In general, an LRS (likelihood ratio statistics) value greater than 17.7 was significant (P genome-wide <0.05) whereas a value between 12 and 17.7 was suggestive. For this study we focused on suggestive loci that were nearly significant and identified in two or more pro-inflammatory cytokine QTL analysis.

### DNA sequencing of candidate genes

Candidate host genes were amplified by PCR using cDNA generated from RNA extracted from HK213 virus-infected lung tissue obtained from B6 and D2 strains. The cDNA was generated using gene-specific primers and Superscript III First-Strand Synthesis System (Invitrogen) according to the manufacturer’s protocol. For a list of primers see Additional file 3: Table S2. Following PCR using the Phusion polymerase protein (Finnzymes), the PCR products were excised from a 1.0% agarose gel and submitted for DNA sequencing (Hartwell Center at St. Jude Children’s Research Hospital) using gene-specific primers. The sequences were aligned and compared to the NCBI reference sequence. The murine candidate genes that were fully sequenced included: *Pon1* (NM_011134.3), *Pon2* (NM_183308.2), *Pon3* (NM_ 173006.1), *Samd9l* (NM_010156.3), *Ica1* (NM_010492.3), *Hepacam2* (NM_178899.5), *Slc25a13* (NM_015829.3)*, Asb4* (NM_023048.5)*, Ccdc132* (NM_024260.5)*, C1galt1* (NM_052993.3). *Col28a1* (NM_001037865.1) was partially amplified and sequenced around exon 16–18 to confirm an in-frame deletion.

### Statistical analysis

Statistical analyses of differences in mortality were determined by using the log-rank test. Differences in morbidity and CCL2 production between control and *Slc25a13*^-/-^ animals were analyzed for statistical significant using Student’s t-test. The Student’s t-test was also used to analyze differences in cytokine concentration in lung homogenates between the different mouse strains or between HK213-infected and mock-infected samples. The Pearson product-moment correlation coefficient was determined for cytokine concentration and virus titer using GraphPad Prism 6 software on untransformed data.

## Electronic supplementary material

Additional file 1: Figure S1: Kinetics of IFN-α production following H5N1 influenza virus infection in DBA/2J and C57BL/6 mice. DBA/2J and C57BL/6J were inoculated with 10^4^ EID_50_ of HK213 virus in 30 μl PBS. Twenty-four, 48 and 72 hours post inoculation the lungs of the inoculated animals were collected, homogenized in sterile PBS, and stored at -80°C. The concentration of IFN-α in these homogenates was quantified by ELISA. (PDF 19 KB)

Additional file 2: Table S1: TNF-α, IFN-α and CCL2 concentrations in lung homogenates of the recombinant inbred BXD animals. BXD mice were inoculated with 10^4^ EID_50_ of HK213 virus in 30 μl PBS. Forty-eight hours post inoculation the lungs of the inoculated animals were collected, homogenized in sterile PBS, and stored at -80°C. The concentration of TNF-α, IFN-α and CCL2 in these homogenates was quantified by ELISA. (PDF 83 KB)

Additional file 3: Table S2: Primer sequences used to amplify the coding region of various candidate genes in *Qivr6.1* locus. (PDF 80 KB)

## References

[1] de Jong MD, Simmons CP, Thanh TT, Hien VM, Smith GJ, Chau TN, Hoang DM, Chau NV, Khanh TH, Dong VC, Qui PT, Cam BV, Ha do Q, Guan Y, Peiris JS, Chinh NT, Hien TT, Farrar J (2006). Fatal outcome of human influenza A (H5N1) is associated with high viral load and hypercytokinemia. Nat Med.

[2] Peiris JS, Yu WC, Leung CW, Cheung CY, Ng WF, Nicholls JM, Ng TK, Chan KH, Lai ST, Lim WL, Yuen KY, Guan Y (2004). Re-emergence of fatal human influenza A subtype H5N1 disease. Lancet.

[3] Horby P, Sudoyo H, Viprakasit V, Fox A, Thai PQ, Yu H, Davila S, Hibberd M, Dunstan SJ, Monteerarat Y, Farrar JJ, Marzuki S, Hien NT (2010). What is the evidence of a role for host genetics in susceptibility to influenza A/H5N1?. Epidemiol Infect.

[4] Olsen SJ, Ungchusak K, Sovann L, Uyeki TM, Dowell SF, Cox NJ, Aldis W, Chunsuttiwat S (2005). Family clustering of avian influenza A (H5N1). Emerg Infect Dis.

[5] Everitt AR, Clare S, Pertel T, John SP, Wash RS, Smith SE, Chin CR, Feeley EM, Sims JS, Adams DJ, Wise HM, Kane L, Goulding D, Digard P, Anttila V, Baillie JK, Walsh TS, Hume DA, Palotie A, Xue Y, Colonna V, Tyler-Smith C, Dunning J, Gordon SB, Everingham K, Dawson H, Hope D, Ramsay P, Campbell A, Walsh Local Lead Investigator TS (2012). IFITM3 restricts the morbidity and mortality associated with influenza. Nature.

[6] Almond MH, Edwards MR, Barclay WS, Johnston SL (2013). Obesity and susceptibility to severe outcomes following respiratory viral infection. Thorax.

[7] Ichinohe T, Pang IK, Kumamoto Y, Peaper DR, Ho JH, Murray TS, Iwasaki A (2011). Microbiota regulates immune defense against respiratory tract influenza A virus infection. Proc Natl Acad Sci U S A.

[8] O'Brien KB, Vogel P, Duan S, Govorkova EA, Webby RJ, McCullers JA, Schultz-Cherry S (2012). Impaired wound healing predisposes obese mice to severe influenza virus infection. J Infect Dis.

[9] Brandes M, Klauschen F, Kuchen S, Germain RN (2013). A systems analysis identifies a feedforward inflammatory circuit leading to lethal influenza infection. Cell.

[10] Hatta Y, Hershberger K, Shinya K, Proll SC, Dubielzig RR, Hatta M, Katze MG, Kawaoka Y, Suresh M (2010). Viral replication rate regulates clinical outcome and CD8 T cell responses during highly pathogenic H5N1 influenza virus infection in mice. PLoS Pathog.

[11] Boon AC, Finkelstein D, Zheng M, Liao G, Allard J, Klumpp K, Webster R, Peltz G, Webby RJ (2011). H5N1 influenza virus pathogenesis in genetically diverse mice is mediated at the level of viral load. MBio.

[12] Pang IK, Pillai PS, Iwasaki A (2013). Efficient influenza A virus replication in the respiratory tract requires signals from TLR7 and RIG-I. Proc Natl Acad Sci U S A.

[13] Boon AC, de Beauchamp J, Hollmann A, Luke J, Kotb M, Rowe S, Finkelstein D, Neale G, Lu L, Williams RW, Webby RJ (2009). Host genetic variation affects resistance to infection with a highly pathogenic H5N1 influenza A virus in mice. J Virol.

[14] Boivin GA, Pothlichet J, Skamene E, Brown EG, Loredo-Osti JC, Sladek R, Vidal SM (2012). Mapping of clinical and expression quantitative trait loci in a sex-dependent effect of host susceptibility to mouse-adapted influenza H3N2/HK/1/68. J Immunol.

[15] Nedelko T, Kollmus H, Klawonn F, Spijker S, Lu L, Hessman M, Alberts R, Williams RW, Schughart K (2012). Distinct gene loci control the host response to influenza H1N1 virus infection in a time-dependent manner. BMC Genomics.

[16] Ferris MT, Aylor DL, Bottomly D, Whitmore AC, Aicher LD, Bell TA, Bradel-Tretheway B, Bryan JT, Buus RJ, Gralinski LE, Haagmans BL, McMillan L, Miller DR, Rosenzweig E, Valdar W, Wang J, Churchill GA, Threadgill DW, McWeeney SK, Katze MG, Pardo-Manuel de Villena F, Baric RS, Heise MT (2013). Modeling host genetic regulation of influenza pathogenesis in the collaborative cross. PLoS Pathog.

[17] Alberts R, Lu L, Williams RW, Schughart K (2011). Genome-wide analysis of the mouse lung transcriptome reveals novel molecular gene interaction networks and cell-specific expression signatures. Respir Res.

[18] Sinasac DS, Crackower MA, Lee JR, Kobayashi K, Saheki T, Scherer SW, Tsui LC (1999). Genomic structure of the adult-onset type II citrullinemia gene, SLC25A13, and cloning and expression of its mouse homologue. Genomics.

[19] Kobayashi K, Sinasac DS, Iijima M, Boright AP, Begum L, Lee JR, Yasuda T, Ikeda S, Hirano R, Terazono H, Crackower MA, Kondo I, Tsui LC, Scherer SW, Saheki T (1999). The gene mutated in adult-onset type II citrullinaemia encodes a putative mitochondrial carrier protein. Nat Genet.

[20] Palmieri L, Pardo B, Lasorsa FM, del Arco A, Kobayashi K, Iijima M, Runswick MJ, Walker JE, Saheki T, Satrustegui J, Palmieri F (2001). Citrin and aralar1 are Ca(2+)-stimulated aspartate/glutamate transporters in mitochondria. EMBO J.

[21] Saheki T, Kobayashi K, Iijima M, Horiuchi M, Begum L, Jalil MA, Li MX, Lu YB, Ushikai M, Tabata A, Moriyama M, Hsiao KJ, Yang Y (2004). Adult-onset type II citrullinemia and idiopathic neonatal hepatitis caused by citrin deficiency: involvement of the aspartate glutamate carrier for urea synthesis and maintenance of the urea cycle. Mol Genet Metab.

[22] Bradel-Tretheway BG, Mattiacio JL, Krasnoselsky A, Stevenson C, Purdy D, Dewhurst S, Katze MG (2011). Comprehensive proteomic analysis of influenza virus polymerase complex reveals a novel association with mitochondrial proteins and RNA polymerase accessory factors. J Virol.

[23] Liu J, Wennier S, Zhang L, McFadden G (2011). M062 is a host range factor essential for myxoma virus pathogenesis and functions as an antagonist of host SAMD9 in human cells. J Virol.

[24] Jiang Q, Quaynor B, Sun A, Li Q, Matsui H, Honda H, Inaba T, Sprecher E, Uitto J (2011). The Samd9L gene: transcriptional regulation and tissue-specific expression in mouse development. J Invest Dermatol.

[25] Li J, Ding SC, Cho H, Chung BC, Gale M, Chanda SK, Diamond MS (2013). A short hairpin RNA screen of interferon-stimulated genes identifies a novel negative regulator of the cellular antiviral response. MBio.

[26] Nagamachi A, Matsui H, Asou H, Ozaki Y, Aki D, Kanai A, Takubo K, Suda T, Nakamura T, Wolff L, Honda H, Inaba T (2013). Haploinsufficiency of SAMD9L, an endosome fusion facilitator, causes myeloid malignancies in mice mimicking human diseases with monosomy 7. Cancer Cell.

[27] Bailey CC, Huang IC, Kam C, Farzan M (2012). Ifitm3 limits the severity of acute influenza in mice. PLoS Pathog.

[28] Gordon TP, Cavill D, Neufing P, Zhang YJ, Pietropaolo M (2004). ICA69 autoantibodies in primary Sjogren's syndrome. Lupus.

[29] The Blue Mountains Eye Study (BMES) (2013). Genome-wide association study of intraocular pressure identifies the GLCCI1/ICA1 region as a glaucoma susceptibility locus. Hum Mol Genet.

[30] Buffa L, Fuchs E, Pietropaolo M, Barr F, Solimena M (2008). ICA69 is a novel Rab2 effector regulating ER-Golgi trafficking in insulinoma cells. Eur J Cell Biol.

[31] Cao M, Xu J, Shen C, Kam C, Huganir RL, Xia J (2007). PICK1-ICA69 heteromeric BAR domain complex regulates synaptic targeting and surface expression of AMPA receptors. J Neurosci.

[32] Grebe KM, Takeda K, Hickman HD, Bailey AL, Embry AC, Bennink JR, Yewdell JW (2010). Cutting edge: sympathetic nervous system increases proinflammatory cytokines and exacerbates influenza A virus pathogenesis. J Immunol.

[33] Tripp RA, Moore D, Winter J, Anderson LJ (2000). Respiratory syncytial virus infection and G and/or SH protein expression contribute to substance P, which mediates inflammation and enhanced pulmonary disease in BALB/c mice. J Virol.

[34] Helyes Z, Elekes K, Sandor K, Szitter I, Kereskai L, Pinter E, Kemeny A, Szolcsanyi J, McLaughlin L, Vasiliou S, Kipar A, Zimmer A, Hunt SP, Stewart JP, Quinn JP (2010). Involvement of preprotachykinin A gene-encoded peptides and the neurokinin 1 receptor in endotoxin-induced murine airway inflammation. Neuropeptides.

[35] Ichinohe T, Pang IK, Iwasaki A (2010). Influenza virus activates inflammasomes via its intracellular M2 ion channel. Nat Immunol.

[36] Iwasaki A, Pillai PS (2014). Innate immunity to influenza virus infection. Nat Rev Immunol.

[37] Veit G, Kobbe B, Keene DR, Paulsson M, Koch M, Wagener R (2006). Collagen XXVIII, a novel von Willebrand factor A domain-containing protein with many imperfections in the collagenous domain. J Biol Chem.

[38] Gong D, Farley K, White M, Hartshorn KL, Benarafa C, Remold-O'Donnell E (2011). Critical role of serpinB1 in regulating inflammatory responses in pulmonary influenza infection. J Infect Dis.

[39] Kennedy RB, Ovsyannikova IG, Pankratz VS, Haralambieva IH, Vierkant RA, Poland GA (2012). Genome-wide analysis of polymorphisms associated with cytokine responses in smallpox vaccine recipients. Hum Genet.

[40] Jagger BW, Wise HM, Kash JC, Walters KA, Wills NM, Xiao YL, Dunfee RL, Schwartzman LM, Ozinsky A, Bell GL, Dalton RM, Lo A, Efstathiou S, Atkins JF, Firth AE, Taubenberger JK, Digard P (2012). An overlapping protein-coding region in influenza A virus segment 3 modulates the host response. Science.

[41] Aldridge JR, Moseley CE, Boltz DA, Negovetich NJ, Reynolds C, Franks J, Brown SA, Doherty PC, Webster RG, Thomas PG (2009). TNF/iNOS-producing dendritic cells are the necessary evil of lethal influenza virus infection. Proc Natl Acad Sci U S A.

[42] Morita M, Kuba K, Ichikawa A, Nakayama M, Katahira J, Iwamoto R, Watanebe T, Sakabe S, Daidoji T, Nakamura S, Kadowaki A, Ohto T, Nakanishi H, Taguchi R, Nakaya T, Murakami M, Yoneda Y, Arai H, Kawaoka Y, Penninger JM, Arita M, Imai Y (2013). The lipid mediator protectin D1 inhibits influenza virus replication and improves severe influenza. Cell.

[43] Walsh KB, Teijaro JR, Wilker PR, Jatzek A, Fremgen DM, Das SC, Watanabe T, Hatta M, Shinya K, Suresh M, Kawaoka Y, Rosen H, Oldstone MB (2011). Suppression of cytokine storm with a sphingosine analog provides protection against pathogenic influenza virus. Proc Natl Acad Sci U S A.

[44] Saheki T, Iijima M, Li MX, Kobayashi K, Horiuchi M, Ushikai M, Okumura F, Meng XJ, Inoue I, Tajima A, Moriyama M, Eto K, Kadowaki T, Sinasac DS, Tsui LC, Tsuji M, Okano A, Kobayashi T (2007). Citrin/mitochondrial glycerol-3-phosphate dehydrogenase double knock-out mice recapitulate features of human citrin deficiency. J Biol Chem.

[45] Sinasac DS, Moriyama M, Jalil MA, Begum L, Li MX, Iijima M, Horiuchi M, Robinson BH, Kobayashi K, Saheki T, Tsui LC (2004). Slc25a13-knockout mice harbor metabolic deficits but fail to display hallmarks of adult-onset type II citrullinemia. Mol Cell Biol.

[46] Hoffmann E, Neumann G, Hobom G, Webster RG, Kawaoka Y (2000). “Ambisense” approach for the generation of influenza A virus: vRNA and mRNA synthesis from one template. Virology.

[47] Hoffmann E, Neumann G, Kawaoka Y, Hobom G, Webster RG (2000). A DNA transfection system for generation of influenza A virus from eight plasmids. Proc Natl Acad Sci U S A.

[48] Chesler EJ, Lu L, Shou S, Qu Y, Gu J, Wang J, Hsu HC, Mountz JD, Baldwin NE, Langston MA, Threadgill DW, Manly KF, Williams RW (2005). Complex trait analysis of gene expression uncovers polygenic and pleiotropic networks that modulate nervous system function. Nat Genet.

[49] Peirce JL, Lu L, Gu J, Silver LM, Williams RW (2004). A new set of BXD recombinant inbred lines from advanced intercross populations in mice. BMC Genet.

[50] Dupuis J, Siegmund D (1999). Statistical methods for mapping quantitative trait loci from a dense set of markers. Genetics.

[51] Shete S, Beasley TM, Etzel CJ, Fernandez JR, Chen J, Allison DB, Amos CI (2004). Effect of winsorization on power and type 1 error of variance components and related methods of QTL detection. Behav Genet.

