# Supplementary figures and images for: A novel genetic locus linked to pro-inflammatory cytokines after virulent H5N1 virus infection in mice
Source: BMC Genomics. 2014 Nov 24;15(1):1017. doi: 10.1186/1471-2164-15-1017 (PMC4256927; doi:10.1186/1471-2164-15-1017)

**Supplemental Figure 1**

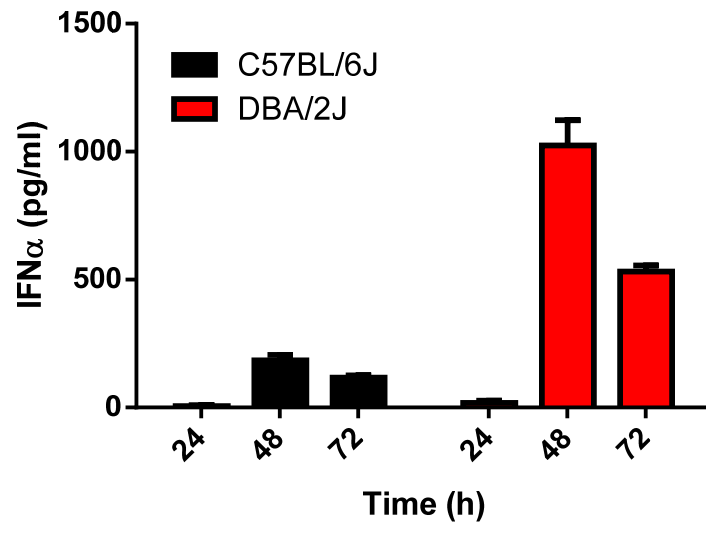

Supplement: Supplementary file 1 — Additional file 1: Figure S1: Kinetics of IFN-α production following H5N1 influenza virus infection in DBA/2J and C57BL/6 mice. DBA/2J and C57BL/6J were inoculated with 104 EID50 of HK213 virus in 30 μl PBS. Twenty-four, 48 and 72 hours post inoculation the lungs of the inoculated animals were collected, homogenized in sterile PBS, and stored at -80°C. The concentration of IFN-α in these homogenates was quantified by ELISA. (PDF 19 KB) [file 12864_2014_6714_MOESM1_ESM.pdf]
