# Supplementary material for: A novel genetic locus linked to pro-inflammatory cytokines after virulent H5N1 virus infection in mice
Source: BMC Genomics. 2014 Nov 24;15(1):1017. doi: 10.1186/1471-2164-15-1017 (PMC4256927; doi:10.1186/1471-2164-15-1017)
Supplement: Supplementary file 2 — Additional file 2: Table S1: TNF-α, IFN-α and CCL2 concentrations in lung homogenates of the recombinant inbred BXD animals. BXD mice were inoculated with 104 EID50 of HK213 virus in 30 μl PBS. Forty-eight hours post inoculation the lungs of the inoculated animals were collected, homogenized in sterile PBS, and stored at -80°C. The concentration of TNF-α, IFN-α and CCL2 in these homogenates was quantified by ELISA. (PDF 83 KB) [file 12864_2014_6714_MOESM2_ESM.pdf]

Supplemental Table 1

Supplemantal Data: CCL2, TNFα, IFN-α concentrations in lung homogenates (pg/ml)												
Jacco Boon and Richard Webby												
Date: 09-12-2013												
	# mice	CCL2	SD	SEM	# mice	TNFa	SD	SEM	# mice	IFNa	SD	SEM
**BXD100***	4	1627	578	289	4	27	9	4	4	412	94	47
**BXD12**	2	5609	2480	1754	2	183	104	74	2	537	369	261
**BXD13**	4	6128	869	434	4	148	15	7	4	494	48	24
**BXD14**	4	4365	618	309	4	85	17	8	4	868	15	7
**BXD18**	4	2064	100	50	4	58	20	10	4	279	95	48
**BXD22**	4	5453	2126	1063	4	165	16	8	4	703	194	97
**BXD27**	4	6068	1138	569	4	233	52	26	4	844	30	15
**BXD28**	4	2945	393	196	4	100	19	10	4	495	25	12
**BXD31**	6	2134	977	399	6	55	42	17	3	368	67	38
**BXD32**	7	4504	1056	399	7	102	13	5	7	616	249	94
**BXD43**	5	1548	535	239	5	37	15	7	10	348	76	24
**BXD44**	7	4632	1112	420	7	191	44	17	7	789	96	36
**BXD45**	5	3341	673	301	5	73	28	12	5	353	114	51
**BXD48**	4	3503	1029	515	4	97	30	15	3	748	180	104
**BXD50**	4	3961	221	111	4	207	19	10	4	844	60	30
**BXD51***	4	1147	280	140	4	11	9	5	4	186	33	16
**BXD55**	8	2744	404	143	8	79	15	5	5	397	68	30
**BXD56**	9	2195	808	269	9	77	40	13	9	248	79	26
**BXD6**	3	3880	1157	668	3	83	44	25	3	716	84	49
**BXD60**	5	2637	654	293	5	179	26	12	5	652	29	13
**BXD61***	7	2181	427	161	7	45	14	5	4	411	78	39
**BXD62***	4	5063	1049	525	4	180	21	10	4	651	10	5
**BXD63**	5	3343	808	361	5	131	24	11	5	545	99	44
**BXD65**	3	2554	334	193	3	51	10	6	3	418	112	65
**BXD66**	5	1611	253	113	5	51	16	7	4	159	79	39
**BXD67**	3	1703	383	221	3	88	9	5	9	514	145	48
**BXD68**	8	1845	704	249	8	46	11	4	9	230	80	27
**BXD69**	6	5081	1413	577	6	133	43	18	8	635	164	58
**BXD70***	4	2040	364	182	4	29	10	5	4	274	82	41
**BXD71**	5	728	228	102	5	15	9	4	5	127	46	21
**BXD73***	8	4125	1300	460	8	118	40	14	8	594	265	94
**BXD75**	5	2523	747	334	5	21	5	2	5	501	49	22
**BXD80**	5	2360	464	207	5	44	11	5	5	199	37	17
**BXD83**	5	1005	269	120	5	34	3	2		-	-	-
**BXD84***	4	4348	592	296	4	120	35	18	4	623	28	14
**BXD85**	3	1173	236	136	3	39	18	10	3	199	92	53
**BXD86**	4	2986	716	358	4	269	30	15	4	677	32	16
**BXD87***	7	3081	860	325	7	63	34	13	3	628	5	3
**BXD89**	9	2668	712	237	9	53	21	7	8	481	149	53
**BXD9**	4	4147	578	289	4	168	22	11	4	982	25	13
**BXD92**	7	1503	392	148	7	25	12	5	7	395	156	59
**BXD96**	9	2364	962	321	9	77	27	9	8	531	193	68
**BXD98**	7	2024	462	174	7	126	63	24	5	525	101	45
**BXD99**	6	3098	661	270	6	78	19	8	2	358	2	1
**C57BL/6J**	15	1982	519	134	15	21	13	3	11	185	70	21
**DBA/2J**	13	8270	1656	459	13	301	58	16	11	1024	328	99
**C57BL/6J Mock infected**	4	42	48	24	4	0	0	0	4	2	1	0
**DBA/2J Mock infected**	4	60	15	8	4	0	0	0	4	1	0	0
