# Supplementary material for: A novel genetic locus linked to pro-inflammatory cytokines after virulent H5N1 virus infection in mice
Source: BMC Genomics. 2014 Nov 24;15(1):1017. doi: 10.1186/1471-2164-15-1017 (PMC4256927; doi:10.1186/1471-2164-15-1017)
Supplement: Supplementary file 3 — Additional file 3: Table S2: Primer sequences used to amplify the coding region of various candidate genes in Qivr6.1 locus. (PDF 80 KB) [file 12864_2014_6714_MOESM3_ESM.pdf]

Supplemental Table 2

Primer Name	Nucleotide Sequence
mAsb4 Fw primer	CATCACTGCCCCTATCA
mAsb4 Fw sequence primer	TTGGGTTGGTTATCACACTTTAGG
mAsb4 Rev primer	CAGTACTCCGTGCTTTGTCT
mAsb4 Rev sequence primer	GCCGTGTGCAGGGGTGTC
mAsb4 Rev sequence primer	CAAGCTTAAGCGGTCACTCACAAA
mAsb4 Rev sequence primer	CAACGGCAGTGGGAGGGACAG
mC1galt1 Fw primer	TCCCTGCCCTTCGTTGAC
mC1galt1 Rev primer	AGTGCCCTTCTTATTCTTCTTGA
mCcdc132 Fw primer	GGATTATGTGATTTGTTACTTCTC
mCcdc132 Fw primer	ATCTGCACAAATGGGAGGAG
mCcdc132 Fw primer	TGCCCTGTTAAGTCAAAT
mCcdc132 Rev primer	AGCCACTATCCCTTCCTTCTATGT
mCcdc132 Rev sequence primer	ACTGATGGGGAGCGAGACTGTT
mCcdc132 Rev sequence primer	GCAGGCATCACAGCATC
mCcdc132 Rev sequence primer	ACAGGCAGAGGTATCCGAACT
mHepacam Fw sequence primer	AGGGGAATGGAACTCTGTCTGC
mHepacam Fw sequence primer	TGAGAAAGTGGCCCAGAAGACAGC
mHepacam Fw sequence primer	GGTCTTCCCCTGCCTCTGA
mHepacam Rev sequence primer	TCGCGTCTTCCGGTCATGTT
mHepacam Rev sequence primer	ACCATCAGAGGCAGGGGAAGACC
mHepacam Rev sequence primer	AAAGCTTATTGCGAACATCTACCA
mHepacam2 Fw primer	ATGGGACAGGATGCTTTTAT
mHepacam2 Rev primer	AACCATTTTGCCTGTCTTTC
mIca1 Fw primer	TGATCCGCCCCAAACCA
mIca1 Rev primer	AGCACACTGAGCATATTAGACTT
mIca1 seq primer	GGCAGTTAATTTCTTTGGAGGATG
mIca1 seq primer	AGAACCTGAAAGTGGGGACAA
mIca1 seq primer	GACAAGTCCAGGCAGGTTCTCT
mIca1 seq primer	CACGGGGAAGAAGGAAGATGAACA
mIca1 seq primer	TGTTGTGGATGAAGGATGTGTCTC
mIca1 seq primer	ACGCTTGTTCCCTCTTCAGTCTT
mIca1 Fw primer	GTCCAAACGCCAGATACCC
mIca1 Rev primer	TACCAGGAACCGGATAGAGAA
mIca1-Exon13 PCR primers Fw	ATCAGCCTCGAGACAGCTCC
mIca1-Exon13 PCR primers Rev	TTCTGTATTAAAGGGGTGGGG
mPon1 Fw primer	TGTTGCTGCACTTGTCC
mPon1 Rev primer	AATAATGGCCTCACTTTC
mPon2 Fw primer	CCGGATGGTGGCTCTGA
mPon2 Rev primer	CTCCCCGGATTTTGATTAGTCT
mPon2 Rev Seq primer	GAGCTCGTGTTTAATTGTTTTCAG
mPon2 Seq primer	CACTACTTCTCCGACCCTTTCTTG
mPon2 Seq primer	TGTACCACCCGAACCATCCTC
mPon3 Fw primer	TTAATAGGGGAAAGGCTGCTGAA

Primer Name	Nucleotide Sequence
mPon3 Rev primer	CGGTCGGAGGTGGTTTTAGTGAA
mSamd9l 3' Rev primer	TATGGGCTAGAAAGAGTAAGTA
mSamd9l 3' Fw primer	AGAAGTTTCGGTGTGCTGTC
mSamd9l 3' Fw sequence primer	TTTTTGGGAATCACTTACACC
mSamd9l 3' Fw sequence primer	GATGATCTGGCCTATTTCCTA
mSamd9l 3' Rev sequence primer	CTTGGCTGCCTGGACCTACACA
mSamd9l 3' Rev sequence primer	GCTGGGCCCTCCTGCTTTTA
mSamd9l 5' Rev primer	ATCGCCAACGTAGTACCTCCACAG
mSamd9l 5' Fw primer	CTCTTGCCAGCATTAGCCACACC
mSamd9l 5' Fw sequence primer	ACGGGGAAATTGTTGGTGTG
mSamd9l 5' Fw sequence primer	AGACAGGGCCACTCAATC
mSamd9l 5' Fw sequence primer	CTCTAAGAATATCCTGGGCAATCC
mSamd9l 5' Fw sequence primer	AAGGGAAGGTTTTGGTGGTGT
mSamd9l 5' Rev sequence primer	AATTTCCCCGTGTGGTTTGT
mSamd9l 5' Rev sequence primer	TCTTTGTTTGCTGTTCTTTTGTTG
mSlc25a13 Fw primer	TCACCGCCCCCACCTCT
mSlc25a13 Fw primer + EcoRI site	TGAATTCTCACCGCCCCCACCTCT
mSlc25a13 Genotying long arm	GATCTACCAAACTACCTTTGCAGAC
mSlc25a13 Genotyping Exon 10	CTTTCTTCTGCAGCTCGCAGAGTC
mSlc25a13 Rev primer	ATAATTCCCCATGATCTTGCTACA
mSlc25a13 Rev primer	ATGGGACGGATGGTGAC
mSlc25a13 Rev primer	AGCCGGTTGATCTCTGGTTCTGC
mSlc25a13 Rev primer	AGGCTTCCGGGGCTGACC
mSlc25a13 Rev primer	CAGACCCAGATGAGGCTTATTGTG
mSlc25a13 Rev primer + AgeI Restriction-site	TACCGGTATAATTCCCCATGATCTTGCTACA
mSlc25a13 Genotyping Neo-Primer	GAAGGAGCAAAGCTGCTATTGGC
